# Supplementary material for: Myocardial global longitudinal strain: An early indicator of cardiac interstitial fibrosis modified by spironolactone, in a unique hypertensive rat model
Source: PLoS One. 2019 Aug 12;14(8):e0220837. doi: 10.1371/journal.pone.0220837 (PMC6690508; doi:10.1371/journal.pone.0220837)
Supplement: S1 Fig — (DOCX) [file pone.0220837.s002.docx]

**S1 fig: Calculation of total cardiac area.**

| 9mm from the apex | 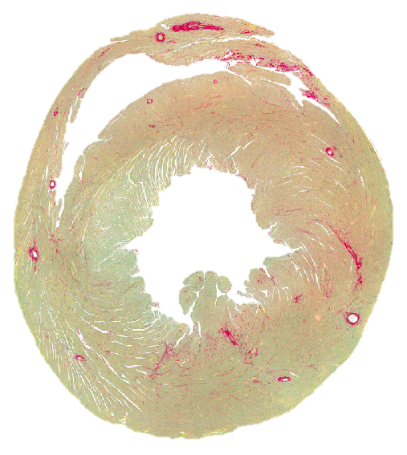  r_2_  r_1_ |  |  |
| --- | --- | --- | --- |
| 6mm from the apex | 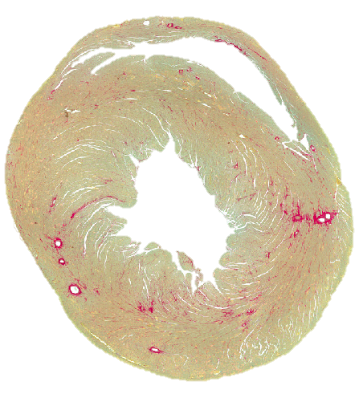 |  |  |
| 3mm from the apex | 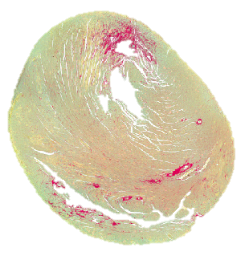 |  |  |

To calculate the total area (volume), each section of heart (cut at 3mm intervals from the apex) had four radial measurements of the inside of the ventricle (r_1_, black arrow), and four more of the total ventricle (r_2_, red arrow). The means were calculated before subtracting r_1_ from r_2._  Each section was assumed to be a cylinder shape with a height of 3mm, and a total volume calculated for each section before being totalled.
