# Supplementary material for: Myocardial global longitudinal strain: An early indicator of cardiac interstitial fibrosis modified by spironolactone, in a unique hypertensive rat model
Source: PLoS One. 2019 Aug 12;14(8):e0220837. doi: 10.1371/journal.pone.0220837 (PMC6690508; doi:10.1371/journal.pone.0220837)
Supplement: S1 Table — (DOCX) [file pone.0220837.s003.docx]

**S1Table. Total cardiac area and fibrosis compared to single mid-section (6mm from apex).**

|  | **N** | **H (one month)** | **H (three month)** |
| --- | --- | --- | --- |
| **Total estimate cardiac area (mm^3^)** | 64.9±1.2 | 68.1±0.0 | 73.3±3.7 |
| **Total estimate fibrosis (%)** | 1.9±0.1 | 3.2±0.1 | 6.9±0.5 |
| **6mm section fibrosis (%)** | 2.4±0.1 | 4.2±1.5 | 7.5±1.1 |

Total area (volume) estimates (n=2) and total fibrosis estimates for normotensive, and hypertensive groups (both after one month and three months). To estimate the total percentage of fibrosis in each section, 5 selections (at 50x mag) from the myocardial layer were captured, and a pixel classifier used to calculate the total percentage of fibrosis. This was then averaged for each section to give a total estimate, before being totalled for all three heart sections. Assuming that the fibrosis distribution was consistent throughout teach section, the total average percentage of fibrosis was used to calculate the total area (volume) of fibrosis in each heart. Finally, these estimates of total volume of fibrosis were compared to the fibrosis calculations obtained (using pixel classifier) using only the section taken 6mm from the apex for each group (10 selections from the myocardium, from each animal, at 50x mag). Values are shown as mean ± standard deviation.

The fibrosis calculated in each method is similar, and in the interests in time, number of assumptions and accuracy, we chose to use the later method for the remainder of this research.
