# Supplementary material for: Myocardial global longitudinal strain: An early indicator of cardiac interstitial fibrosis modified by spironolactone, in a unique hypertensive rat model
Source: PLoS One. 2019 Aug 12;14(8):e0220837. doi: 10.1371/journal.pone.0220837 (PMC6690508; doi:10.1371/journal.pone.0220837)
Supplement: S2 Table — (DOCX) [file pone.0220837.s004.docx]

**S2 table. Left ventricular ejection fraction obtained by different standard measurement techniques**.

|  |  | **M-Mode (%)** | **2D “bullet” (%)** | **TomTec 2D (%)** |
| --- | --- | --- | --- | --- |
| **One month** | **N** | 91±1 | 83±2 | 84±3 |
|  | **H** | 92±6 | 85±3 | 81±1 |
|  | **H+SP** | 95±2 | 78±7 | 83±2 |
|  | **R^2 a^** | *0.002* | *0.2* | *0.37** |
|  |  |  |  |  |
| **Three months** | **N** | 92±2 | 83±1 | 80±3 |
|  | **H** | 88±6 | 77±4 | 67±7 |
|  | **H+SP** | 88±10 | 82±5 | 72±4 |
|  | **R^2^** | *0.02* | *0.15* | *0.37*** |

Left ventricular ejection fraction (LVEF) obtained by different standard measurement techniques (n=4-8), M-mode measurements and longitudinal 2-dimensional (2D) algorithm (“bullet”, which utilises the area/volume and the apex to mitral valve measurement at both diastole and systole) using the EchoPac software (EchoPac, version.112.0.x, GE Healthcare, USA), and the LVEF obtained using longitudinal 2D images in the Tom Tec software (TomTec Image-Arena, version 2.21; TomTec Imaging Systems, Unterschleissheim, Germany). All three standard LVEF measurements were correlated to fibrosis scores (%) for the same animal at each time point. Linear correlations (R^2^) between myocardial fibrosis and LVEF were much stronger with the use of the 2D analysis using the TomTec software that that of the more standardised M-mode or the 2D “bullet” algorithm used in EchoPac’s software. Significant differences between fibrosis scores and EF method is indicated by *, p<0.05 ^*^, p<0.01 ^**^
