# Supplementary material for: Myocardial global longitudinal strain: An early indicator of cardiac interstitial fibrosis modified by spironolactone, in a unique hypertensive rat model
Source: PLoS One. 2019 Aug 12;14(8):e0220837. doi: 10.1371/journal.pone.0220837 (PMC6690508; doi:10.1371/journal.pone.0220837)
Supplement: Attachment — Submitted filename: PLos One response.docx [file pone.0220837.s005.docx]

PLOS One response:

ACADEMIC EDITOR: All issues raised by expert reviewers are required. The authors should limit speculations and highlight limitations of the study.

Response: Comments to the reviewers comments are detailed below. Changes made to manuscript both in PLOS ONE style as well as where requested.

Please include the following items when submitting your revised manuscript:

- A rebuttal letter that responds to each point raised by the academic editor and reviewer(s). This letter should be uploaded as separate file and labeled 'Response to Reviewers'.
- A marked-up copy of your manuscript that highlights changes made to the original version. This file should be uploaded as separate file and labeled 'Revised Manuscript with Track Changes'.
- An unmarked version of your revised paper without tracked changes. This file should be uploaded as separate file and labeled 'Manuscript'.

1. When submitting your revision, we need you to address these additional requirements.

Please ensure that your manuscript meets PLOS ONE's style requirements.
manuscript has been appropriately modified to PLOS ONE style.

Please include captions for your Supporting Information files at the end of your manuscript, and update any in-text citations to match accordingly including those for file naming.
Supporting information file now in correct format.

We noticed you have some minor occurrence of overlapping text with the following previous publication(s), which needs to be addressed:

<https://www.ingentaconnect.com/content/aalas/cm/2018/00000068/00000005/art00005>

In your revision ensure you cite all your sources (including your own works), and quote or rephrase any duplicated text outside the methods section. Further consideration is dependent on these concerns being addressed.
We have increased the referencing to our own work (reference 18) in the methods section where we can see there may be some duplicated text.

To comply with PLOS ONE submissions requirements, please provide methods of sacrifice in the Methods section of your manuscript.
Animals were terminated by halothane overdose and cardiac puncture. - added

Data availability issue. In your statement you say "All relevant data are within the paper and its Supporting Information files", but as we explain in <http://journals.plos.org/plosone/s/data-availability#loc-faqs-for-data-policy> you should provide the individual data points behind means, medians and variance measures presented in the results, tables and figures, and not just those summary statistics.
We have provided the raw data as requested. As this is animal data it is not clear where this should be deposited, so have provided the raw data as an XCEL file under supporting information.

5. Review Comments to the Author

Please use the space provided to explain your answers to the questions above. You may also include additional comments for the author, including concerns about dual publication, research ethics, or publication ethics. (Please upload your review as an attachment if it exceeds 20,000 characters)

Reviewer #1: This article investigates the effect of spironolactone on cardiac function and fibrosis after induction of hypertension in a transgenic hypertensive rat model. The authors perfomes an advanced echocardiograpic evaluation assessing global longitudinal strain (myocardial and endocardial) that usually is not evaluated in the animal model.
As the authors report GLS can give more information in particular in HFpEF where ejection fraction is preserved.
The article is well written and the topic is relevant in clinical setting.
There are some suggestions:
- the authors should consider to add echocardiographic data of mass in Table 1:
- the discussion section could be a little bit shortened
- the are two figure 1 and no figure 2
- there are two figure 5 and no figure 4.

Response: We thank the reviewer for her helpful comments. LV mass was considered but as this is also a derived calculation, it did not add any additional information, to what was already observed. So it has not been added. The discussion has been shortened where possible (see track change version). The incorrect labelling of the figures has been changed.

Reviewer #2: This experimental study using a hypertensive rat model showed the reduction of myocardial fibrosis with spironolactone, showing the changes in left ventricular ejection fraction and longitudinal strain from speckle tracking echo.

However, the findings do not provide novel insights as compared to previous studies referred.

We would like to disagree with the reviewer. There are several important novel insights. Firstly we have reported the strong correlation between myo-GLS and the degree of cardiac interstitial fibrosis as seen histologically, confirming it as an important non-invasive means to assess cardiac fibrosis. This has not previously been reported.

It is important to clearly define a normal range for ejection fraction in an animal model and not rely on a human range. We believe this is the first time it has been defined for an animal model. This also highlights the potential for this animal model as a model for HFpEF as identified by reviewer 1.

Previous intervention studies have administered spironolactone (or other drugs) at the onset of hypertension, which does not reflect the clinical scenario where patients present with established hypertension and left ventricular hypertrophy. Our model more accurately reflects this. We used a dose for the rats equivalent to the standard human dose. Previous animal studies with spironolactone use supra-physiological doses (often 10 to 100 fold higher than what would be an equivalent human dose) and these are administered either subcutaneously (osmotic pumps) or intra-peritoneally, when spironolactone has no evidence for efficacy other than by oral administration as was used in this study.


Comments

1. The title does not imply the aim and results exactly. The effect of spironolactone should be reflected in the title.

Thank you for this. The title has been changed appropriately.

2. Myo-GLS is more sensitive to the myocardial fibrosis, as shown in Fig 5. However, in the mid portion of the myocardium, cardiac fiber may be located in the circumferential positions rather than longitudinal positions. Please explain the discrepancy between functional and pathological behaviors.
Myocardial global longitudinal strain measures contractile activity in real-time, and cardiac fibers are not static, rather they contract in a 3 dimensional response. This is picked up and allowed for in the global longitudinal strain analysis. Therefore any alterations in contractility related to fibrosis would still be observed.

3. In addition, the transmural distribution of fibrosis is needed to conclude your findings.

Serial sections were analysed for the extent of fibrosis and we focused on the myocardial component rather than including epi and endocardial regions as these were too small to be of significance. Figure 3 demonstrates the regions of interest.

4. This technique may have limitations in reproducibility of strain measurements. The intra- and inter-observers’ variabilities should be clarified.

The echo analyses were undertaken blinded by two of the investigators (MM, SC). The inter-observer and intra-observer interclass variability correlation coefficients were 96% and 97% respectively.
